# Tumor gene therapy by systemic delivery of plasmid DNA with cell‐penetrating peptides

**DOI:** 10.1096/fba.1026

**Published:** 2018-11-26

**Authors:** Kadri Künnapuu, Kadi‐Liis Veiman, Ly Porosk, Evelin Rammul, Kristina Kiisholts, Ülo Langel, Kaido Kurrikoff

**Affiliations:** ^1^ Institute of Technology University of Tartu Tartu Estonia; ^2^ Department of Biochemistry and Biophysics, The Arrhenius Laboratories for Natural Sciences Stockholm University Stockholm Sweden

**Keywords:** cell‐penetrating peptides, gene delivery, nonviral gene therapy, self‐assembly, tumor therapy

## Abstract

Gene therapy is a prospective strategy for treating cancer. However, finding efficient and tumor‐specific gene delivery vectors remains an issue. Tumor responsive cell‐penetrating peptide (CPP) PepFect144 (PF144) has previously been shown to deliver reporter gene encoding plasmid DNA specifically into tumors upon systemic administration, but its capability to reduce tumor growth has not yet been evaluated. Here, we study the potential of PF144‐based anti‐angiogenic gene delivery to inhibit tumor growth by silencing vascular endothelial growth factor (VEGF) expression in tumors. This approach led to the inhibition of tumor growth in both the HT1080 fibrosarcoma model and orthotopic 4T1 breast tumor model. We additionally saw that the addition of αvβ3 integrin targeting did not further improve the tumor sensitive CPPs. Our results suggest that activatable cell‐penetrating peptide PF144 is a promising nonviral plasmid DNA delivery vector for cancer treatment.

## 1 INTRODUCTION

Cancer is one of the leading causes of death worldwide, with the number of new cases expected to rise in the next two decades. The disease is characterized by genetic abnormalities, with 125 cancer‐promoting genes having been found so far, making gene therapy a prospective way to treat cancer.^1^ Gene therapy involves the genetic modification of a patient's cells by transferring genes, gene segments, or regulatory oligonucleotides into the cells. It has the potential to be used as a standalone therapy, or combination therapy with cytotoxic and radiation therapy.^2^

Since genetic material cannot cross the cellular barriers by itself, gene delivery vectors are needed. One of the most widely used gene delivery vectors are viral vectors, which have long been used for their high efficiency. However, their high cost, immunogenicity, and difficult production have led to the search of nonviral alternatives.^3^, ^4^ One class of potential alternatives are cell‐penetrating peptides (CPPs), which are a family of relatively short peptides (5‐30 amino acids) that can pass through tissue and cell membranes. They are capable of transporting a wide variety of bioactive cargo into cells that cannot otherwise cross the cell membrane.^5^, ^6^ CPPs are easy to synthesize, modify, and they can be complexed with nucleic acids non‐covalently, simplifying nanoparticle production.

Cell‐penetrating peptides can be PEGylated to increase plasma half‐life.^7^ However, PEGylated nanoparticles have shown to have a slower uptake into tumors than non‐PEGylated particles, necessitating the use of a strategy where the PEG molecule could eventually be cleaved.^8^ Using cleavage sites for tumor‐specific proteases also helps increase tumor specificity. Matrix metalloproteinases 2 and 9 (MMP‐2/‐9) have shown to be overexpressed in many types of tumors, and its cleavage sites have previously been used for the aforementioned purpose.^9^ Upregulation of MMP production during tumor progression is necessary for degrading basement membrane components which allow the tumors to grow, invade surrounding tissue, and metastasize. They are important positive regulators of angiogenesis, and the activity of MMPs has been found to correlate with tumor stage.^10^, ^11^, ^12^

In a previous paper MMP‐activatable CPPs PepFect144 (PF144), PepFect145 (PF145), and PepFect146 (PF146) were designed, where the cell penetration ability of the CPPs was reversibly masked by attaching a PEG molecule to the C‐terminus of the peptides via an MMP‐2/‐9 cleavable linker. These peptides are all based on the CPP PepFect14 (PF14)^13^ and they differ from each other only by the size of the shielding PEG moiety. They are successful in mediating tumor‐specific gene delivery of plasmid DNA encoding a reporter gene.^14^

Tumor angiogenesis is one of the driving forces behind cancer development, and anti‐angiogenic therapy can thus be an effective way to reduce or inhibit the development of tumors as a part of combinatorial treatment.^15^ The inhibition of angiogenesis in an adjuvant setting can prevent relapse,^16^ and as neoadjuvant therapy, the antiangiogenic therapy could be beneficial in shrinking a non‐resectable tumor into one that is potentially operable.^17^ VEGF is a pro‐angiogenic factor which is overexpressed in most solid cancers; downregulating its expression could lead to the inhibition of new blood vessel formation, and an increase in the permeability of the tumor to cytotoxic agents.^18^, ^19^

In this work we aim to test the potential use of the MMP‐2/‐9 activatable CPPs for antitumor therapy upon systemic administration of the nanoparticles consisting of CPPs and plasmid DNA (pDNA) that expresses short hairpin RNA against VEGF (shVEGF) to tumor‐bearing mice. We hypothesize that our delivery method enables the knockdown of VEGF, leading to suppression of tumor angiogenesis, and inhibiting tumor growth. We additionally study the effect of a targeting ligand, the iRGD peptide on the tumor gene delivery efficiency of the MMP‐2/‐9 activatable CPPs.^20^

## 2 MATERIALS AND METHODS

### 2.1 Peptide synthesis

Peptides were synthesized using an automated peptide synthesizer (Biotage, Uppsala, Sweden) in a 0.1 mmol scale by standard protocols for Fmoc solid‐phase synthesis. Rink amide ChemMatrix resin (Biotage) was used as solid phase to obtain C‐terminally amidated peptides. Stearic acid (Sigma‐Aldrich, Munich, Germany) and PEG (PEG1000, polydisperse—Jenkem, Plano, TX; PEG600, monodisperse—Chempep, Wellington, FL) were coupled like standard amino acids, with coupling times of 18 hours and 24 hours, respectively. For the generation of intramolecular disulfide bonds, the peptidyl resin was treated with 1.6 eqv of thallium (III) trifluoroacetate (Sigma‐Aldrich) in DMF (Sigma‐Aldrich) for 30 minutes.^21^ Cleavage from resin was done following standard protocol (95% trifluoroacetic acid [TFA] [Sigma‐Aldrich], 2.5% TIS [Sigma‐Aldrich], 2.5% MQ water). The peptides were purified by RP‐HPLC (Agilent, Santa Clara, CA) using a C3 column and 20%‐90% acetonitrile gradient in water (0.1% TFA). Molecular weights of the peptides were analyzed by MALDI‐TOF mass‐spectrometry, and purities were determined by UPLC (Waters, Milford, MA). Disulfide bond formation was confirmed with Ellmann's test (Ellmann's reagent, Sigma‐Aldrich).

### 2.2 CPP/pDNA complex formation

CPP/pDNA nanoparticles for cell culture assays were prepared by mixing plasmid DNA (0.5 μg per well of a 24‐well plate) with CPPs, at CPP/pDNA charge ratio 3 (CR3) in MQ water with final volume of 105 μL, followed by a 40‐minute incubation at room temperature. pGL3 Control (Promega, Stockholm, Sweden) was used as the luciferase encoding plasmid since it is best suitable for in vitro use.

For in vivo studies, the plasmid dose was 0.8 mg/kg per animal; the pDNA was mixed with the CPP at CR 4 in MQ water, keeping the final complex volume at 100 μL. After 40‐minute incubation, 100 μL of 10% glucose was added to the complexes and injected immediately via tail vein. The luciferase encoding plasmid used in reporter gene experiments in vivo was p‐CMV‐Luc2, marked as pLuc2 in the text, which is optimized for in vivo use.

If not stated otherwise, the PEGylation rate of peptides in the CPP/pDNA complexes was 50% in all in vivo experiments. In this case, 50% of the total peptide content in the complexes is made up of PEGylated CPPs (PF144, PF147, PF145‐iRGD, or PF148‐iRGD), and the other half of PF14, which is not PEGylated. To increase the probability of PEGylated peptides being incorporated into the complex, the pDNA was first incubated with the PEGylated peptide for 5 minutes before adding PF14.

### 2.3 Dynamic light scattering (DLS) measurements

Hydrodynamic mean diameter was measured by dynamic light scattering, using Zetasizer Nano ZS apparatus (Malvern Instruments, Malvern, United Kingdom). For size measurements, CPP/pDNA complexes were formulated according to the protocol for cell culture assays, as described above, at CR4. The solutions containing the complexes were transferred to low volume cuvettes after 40 minutes of incubation, and the size measured. Each sample (n = 2) was measured three times, 12 measurement runs (1 run = 10 seconds) each. For measuring the zeta potential, the complexes were formulated at CR4 as described above, at a total volume of 300 μL, and diluted to 1 mL with MQ water after 40 minutes of incubation. For each sample (n = 2) three measurements of 10 runs were made.

### 2.4 Cell culture

Neuro2a and HT1080 cells were cultured in a humidified environment at 37°C, 5% CO2, and cultivated in Dulbecco's Modified Eagle's Medium (Thermo Fisher Scientific, Uppsala, Sweden) which was supplemented with glutamax, 0.1 mM non‐essential amino acids, 1.0 mM sodium pyruvate, 10% FBS, 100 U/mL penicillin, and 100 μg/mL streptomycin (PAA Laboratories GmbH, Germany). Similarly supplemented RPMI 1640 medium (Thermo Fisher Scientific) was used for cultivating 4T1 cells. All cell culture experiments were performed in the supplemented media.

### 2.5 In vitro transfection

Twenty‐four hours before the experiment 3 × 10^4^ Neuro2a cells were seeded onto 24‐well plates. Peptide/pDNA complexes at CR3 were prepared as described above. The transfection mixture was added to cells containing full media, and it made up 1/10 of the total cell medium volume during the time of transfection. Lipofectamine^™^ 2000 (Invitrogen, Carlsbad, CA) was used according to the manufacturer's protocol and added to the cells in serum‐containing medium. After 4‐hour incubation with the complexes, an additional 1 mL of fresh medium was added to the cells and incubated for an additional 20 hours. Thereafter, the media was removed, cells were lyzed with 100 μL 0.1% Triton X‐100 in PBS for 15 minutes at 4°C. Luciferase activity was measured using Promega's luciferase assay (Luciferase Assay System, Promega) according to the manufacturer's protocol on GLOMAX^™^ 96 microplate luminometer (Promega) and normalized to protein content, which was measured with the BioRad Protein Assay (Hercules, CA) according to the manufacturer's protocol.

To study the effect of MMP‐2 cleavage on transfection efficiency, active recombinant MMP‐2 enzyme (0.1 mg/mL, Calbiochem, Germany) was added to the CPP/pDNA complexes in MQ (final MMP‐2 concentration 3.8 ng/μL), and incubated at 37°C for 40 minutes prior transfection.

### 2.6 Resistance of complexes to degradation in serum

The stability of the CPP/pDNA nanoparticles to degradation and dissociation by components found in serum was assessed by reporter gene expression in cells after 4 hours incubation of CPP/pLuc2 complexes with 50% FBS. Complexes were formed as described previously, with 0.1 μg pDNA (CR 3), and incubated at room temperature for 40 minutes. After this, FBS (or MQ for controls) was added to the complexes, and the mixture incubated at 37°C for 4 hours (final FBS concentration 50%). For transfection, 1 × 10^4^ Neuro2a cells were seeded to transparent 96‐well plates in supplemented DMEM media 24 hours before experiment. On the day of the experiment, the cells were transfected and luciferase expression analyzed as described in previous section (In vitro transfection).

### 2.7 Heparin displacement assay

Accessibility of pDNA in complexes formed between pDNA and CPPs was assessed by Quant‐iT^™^ PicoGreen^®^ (Thermo Fisher Scientific) assay in mQ water. CPP/pLuc2 nanoparticles at CR3 were prepared as described previously, and transferred to black 96 well plates (0.1 μg of pDNA per well). Heparin solution was added into the wells and incubated at 37°C for 30 minutes. For detection, PicoGreen^®^ was added to the wells, and incubated for 20 minutes. Fluorescence was measured by fluorimeter (λ_ex _= 492 nm, λ_em _= 535 nm) (SynergyMx, BioTek).

### 2.8 Design of short hairpin VEGF expressing gene vector

Human VEGF (GenBank M32977.1)‐specific shRNA expressing plasmid was designed and constructed using the backbone of pCpGfree‐siRNA (version 14L08‐MM, Invivogen), according to the manufacturer's instructions. Briefly, two complementary oligonucleotides of siVEGF sequence containing hairpin structure were constructed for the ligation into BbsI‐linearized pCpGfree‐siRNA vector, resulting in pshVEGF. Correct insertion was verified by sequencing. The hairpin sequence is as follows:

5’ AAGGAGTACCCTGATGAGATC TTCAAGAGA GATCTCATCAGGGTACTCCTT tttttt 3’ (sense).

5’ aaaaaa AAGGAGTACCCTGATGAGATC TCTCTTGAA GATCTCATCAGGGTACTCCTT 3’ (antisense).

The VEGF target sequence is shown in uppercase and the loop is underlined.

### 2.9 Tumor induction

All animal experiments and procedures were approved by the Estonian Laboratory Animal Ethics Committee (approvals no 81, Apr 04, 2016, and 69 and 70, February 9, 2011).

Mouse Neuro2a and HT1080 tumor xenografts were induced by resuspending 1 × 10^6^ cells in 100 μL volume of ice‐cold un‐supplemented DMEM and implanting the cell suspension subcutaneously to the right flank of the mouse (Neuro2a: 4‐ to 6‐week‐old BALB/c; HT1080: Hsd:Athymic Nude‐Foxn1nu female, 4‐6 weeks old, Harlan, UK). The 4T1 orthotopic tumors were induced by injecting 5 × 10^4^ cells in 50 μL of ice‐cold un‐supplemented RPMI into the fourth mammary fat pad of 4‐ to 8‐week‐old female BALB/c mice.

### 2.10 Reporter gene delivery assessment in vivo

Twenty‐four hours after the intravenous administration of CPP/pLuc2 complexes, the mice bearing Neuro2a tumors were sacrificed by cervical dislocation and tissues were harvested and snap‐frozen on dry ice. The tissues were homogenized using Precellys^®^24‐Dual homogenization system (Bertin Technologies, France) and lyzed with 1 × Promega lysis buffer (Promega). Luminescence was analyzed as described in in vitro transfection methods section.

### 2.11 Tumor growth reduction studies

Before treatment, the mice were assigned into six groups of 10. CPP/pshVEGF treatment for HT1080 tumor‐bearing animals was started at the first appearance of tumor growth (tumor size of approximately 100 mm^3^). With the 4T1 breast tumor model, the treatment was started 1 day after cancer cell implantation. The first two doses of the CPP/pshVEGF complexes and pshVEGF control (pDNA without CPP) were administered intravenously via tail vein, and the third dose intraperitoneally. The tumor sizes were measured with calipers three times a week. With both the HT1080 and 4T1 model, the experiment was terminated and mice were euthanized at the first occurrence of lethargy, which took place before the tumors could reach the cutoff value (1000 mm^3^).

## 3 RESULTS

### 3.1 The design of iRGD‐functionalized PepFects

In order to study the effect of integrin targeting on the CPPs previously introduced by Veiman et al,^14^ iRGD‐functionalized peptides based on PF144 and PF145 were synthesized. The motif PLGLAG was kept as the MMP‐2/‐9 enzyme cleavage site. The sequences of the iRGD‐modified peptides and their parent CPPs are presented in Table 1. The iRGD sequence was added to the chain terminus of the PEG moiety of the selected CPPs to increase the likelihood of iRGD being located on the surface of the particles. PF148‐iRGD has a scrambled sequence in the MMP‐2 cleavage site and was synthesized as a control peptide. As expected, the addition of the iRGD sequence to the CPPs did not interfere with the cleavability of the MMP‐2 sensitive linker by the MMP‐2 enzyme, and the uncleavable control was resistant to MMP‐2 degradation as indicated by UPLC (Figure S1).

**Table 1:** Names and sequences of the peptides used in the study

Name	PepFect iteration	Sequence
PF14	PepFect14	Stearyl‐AGYLLGKLLOOLAAAALOOLL‐NH_2_
iRGD		C*RGDKGPDC*‐NH_2_
PF144	PepFect144	Stearyl‐AGYLLGKLLOOLAAAALOOLL‐X‐PLGLAG‐PEG_600_‐NH_2_
PF144‐iRGD	PepFect1440	Stearyl‐AGYLLGKLLOOLAAAALOOLL‐X‐PLGLAG‐PEG_600_‐C*RGDKGPDC*‐NH_2_
PF145	PepFect145	Stearyl‐AGYLLGKLLOOLAAAALOOLL‐X‐PLGLAG‐PEG_1000_‐NH_2_
PF145‐iRGD	PepFect1445	Stearyl‐AGYLLGKLLOOLAAAALOOLL‐X‐PLGLAG‐PEG_1000_‐C*RGDKGPDC*‐NH_2_
PF148	PepFect148	Stearyl‐AGYLLGKLLOOLAAAALOOLL‐X‐LALGPG‐PEG_1000_‐NH_2_
PF148‐iRGD	PepFect1448	Stearyl‐AGYLLGKLLOOLAAAALOOLL‐X‐LALGPG‐PEG_1000_‐C*RGDKGPDC*‐NH_2_

O, Ornithine; X, Aminohexanoic acid; *, Intramolecular disulfide bond between marked cysteines

**Figure 1:**
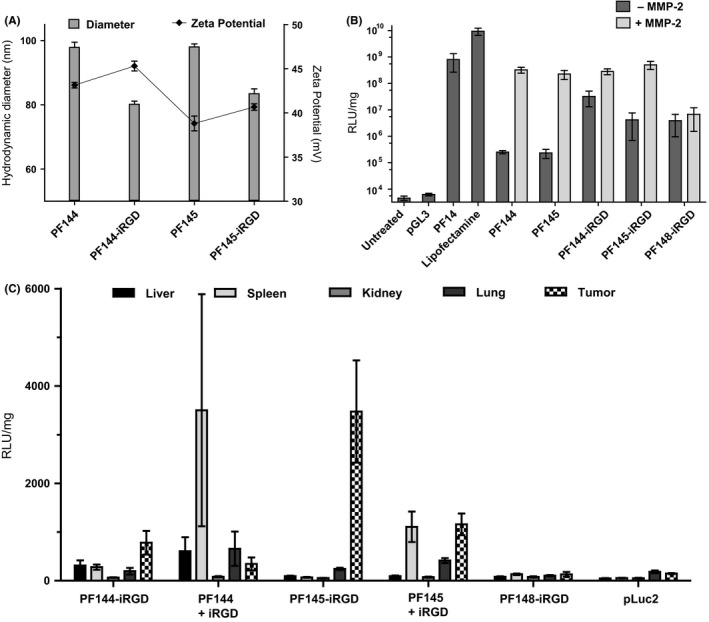
Characterization of iRGD‐decorated CPPs. (A) DLS data of the effect of iRGD conjugation on the hydrodynamic diameter (Z‐average) and zeta potential of the CPP/pDNA complexes. A PEGylation rate of 50% was used in every complex. (B) The effect of MMP‐2 treatment on iRGD‐modified activatable PepFects was evaluated in vitro on Neuro2a cells. CPP/pGL3 complexes at CR3 were added to the Neuro2a cells. Luciferase activity was measured 24 h after transfection, and normalized to total protein content. (C) Reporter gene induction in vivo with iRGD‐modified PepFects. BALB/c mice bearing sc Neuro2a tumors were administered one dose of CPP/pLuc2 complexes at CR4 intravenously via tail vein, plasmid dose 0.8 mg/kg. The PEGylation rate of 50% was used in every complex. The molar amount of coadministered iRGD was equal to that of PF14. Tissues were harvested 24 h after injection, homogenized, and luciferase activity measured. Luciferase activity was normalized to total protein content. All data are presented as mean + SEM

The DLS data showed that conjugation of the iRGD sequence to PF144 and PF145 decreased the hydrodynamic diameter of the CPP/pDNA nanoparticles. A slight increase in zeta potential was also observed, suggesting a change in surface potential of the particle arising from the charges of the iRGD moiety (Figure 1A).

### 3.2 iRGD‐functionalized PF145 retains its tumor specificity

The transfection efficiency of the new CPPs was first assessed by transfecting Neuro2a neuroblastoma cells with CPPs complexed with luciferase encoding plasmid DNA (pGL3). We observed that although the iRGD functionalized peptides were able to transfect the cells, elevated transfection ability was present even in their nonactivated form (Figure 1B), the effect more pronounced with PF144‐iRGD that has a shorter overall PEG chain than PF145‐iRGD. After PF144‐iRGD and PF145‐iRGD complexes with pGl3 had been incubated with the MMP‐2 enzyme, their transfection efficiencies were fully restored to the level of PF14. This validates that after modifying the CPPs with iRGD, their enzyme‐dependent activation is retained. As expected, PF148‐iRGD with a scrambled MMP‐2 cleavage site showed no enzyme‐dependent activation (Figure 1B).

Tumor selectivity of the dual‐targeted peptides was verified by administering CPP/pLuc2 nanoparticles to BALB/c mice bearing subcutaneous Neuro2a tumors. The PEGylation rate of peptides in the nanoparticles containing a PEGylated CPP, was 50% (see the Methods part for the details regarding PEGylation rate). The Neuro2a cells have been previously shown to express αvβ3 integrins.^22^, ^23^ We induced the subcutaneous Neuro2a tumors in BALB/c mice, and proceeded to test the efficiency of the modified CPPs. Consistent with the cell culture results, the addition of the iRGD moiety decreased the tumor specificity of PF144, leading to an increase in luciferase levels in the liver and spleen in animals treated with PF144‐iRGD/pLuc2 nanoparticles (Figure 1C), therefore the PF144‐iRGD construct was excluded from further experiments. Adding the iRGD moiety to PF145, however, preserved its tumor specificity (Figure 1C). Additional PEGylation rates of 40% and 70% were tested for PF145‐iRGD, with 50% being the most effective of the three (Figure S2). Coadministering the standalone iRGD peptide with CPP/pLuc2 nanoparticles, as opposed to covalent attachment of iRGD to the CPP, as was first shown by Sugahara et al^24^ resulted in high luciferase levels in spleen when applied to PepFects. Control peptide PF148‐iRGD was unable to induce luciferase expression in any tissue, as expected.

### 3.3 PEG and iRGD weaken PepFect/pDNA complexes

**Figure 2:**
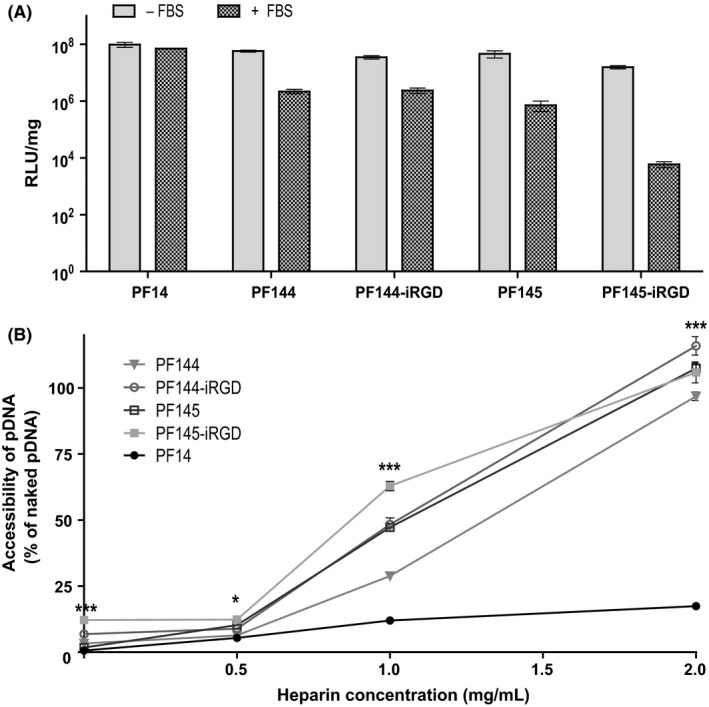
Resistance of CPP/pDNA complexes to degradation. (A) Resistance to serum degradation was assessed by observing differences in transfection efficiency of CPP/pLuc2 complexes in Neuro2a cells before and after a 4‐hour incubation of the nanoparticles in 50% FBS. (B) Heparin displacement assay was used to study the strength of CPP and pDNA interactions in the nanoparticles. Results are represented as relative PicoGreen^®^ fluorescence, where 100% corresponds to the fluorescence of naked pDNA. Stars represent p‐values from two‐way ANOVA with Bonferroni post test between PF144 and PF145‐iRGD groups, *P < 0.05, ***P < 0.001. All data are presented as mean + SEM.

The stability of the CPP/pDNA nanoparticles in the presence of serum is important when the drug is administered via intravenous injection where it comes into contact with different blood components. We assessed this by evaluating the effect of the presence of 50% FBS and heparin on the nanoparticles. After transfecting Neuro2a cells with FBS‐treated CPP/pDNA nanoparticles, a decrease in transfection efficiency proportional to the size and dispersity of PEG and the presence of iRGD can be observed, with PF145‐iRGD being the most, and PF14 being the least sensitive to serum treatment (Figure 2A).

Similar trends can be seen in the heparin displacement assay, where the strength of the CPP‐pDNA interaction was assessed by how easily heparin could displace pDNA from the nanoparticles. PF14 forms the most condense complexes with pDNA, and the pDNA in PF144/pDNA nanoparticles was most resistant to heparin displacement compared to the other PEGylated complexes (Figure 2B).

### 3.4 PF144 mediates effective tumor growth reduction

We proceeded to test whether tumor‐specific PF144 complexed with therapeutically relevant pDNA could reduce tumor growth. Other CPPs included in the study were PF145‐iRGD, and control CPPs PF147 and PF148‐iRGD that are resistant to MMP digestion. The plasmid expression vector for this study was chosen to maximize the production of shRNA in mammalian cells (see the Methods part for the details regarding plasmid construction) and clear knockdown of VEGF expression was verified in HT1080 cells after PF14/pshVEGF transfection in vitro (Figure S3).

**Figure 3:**
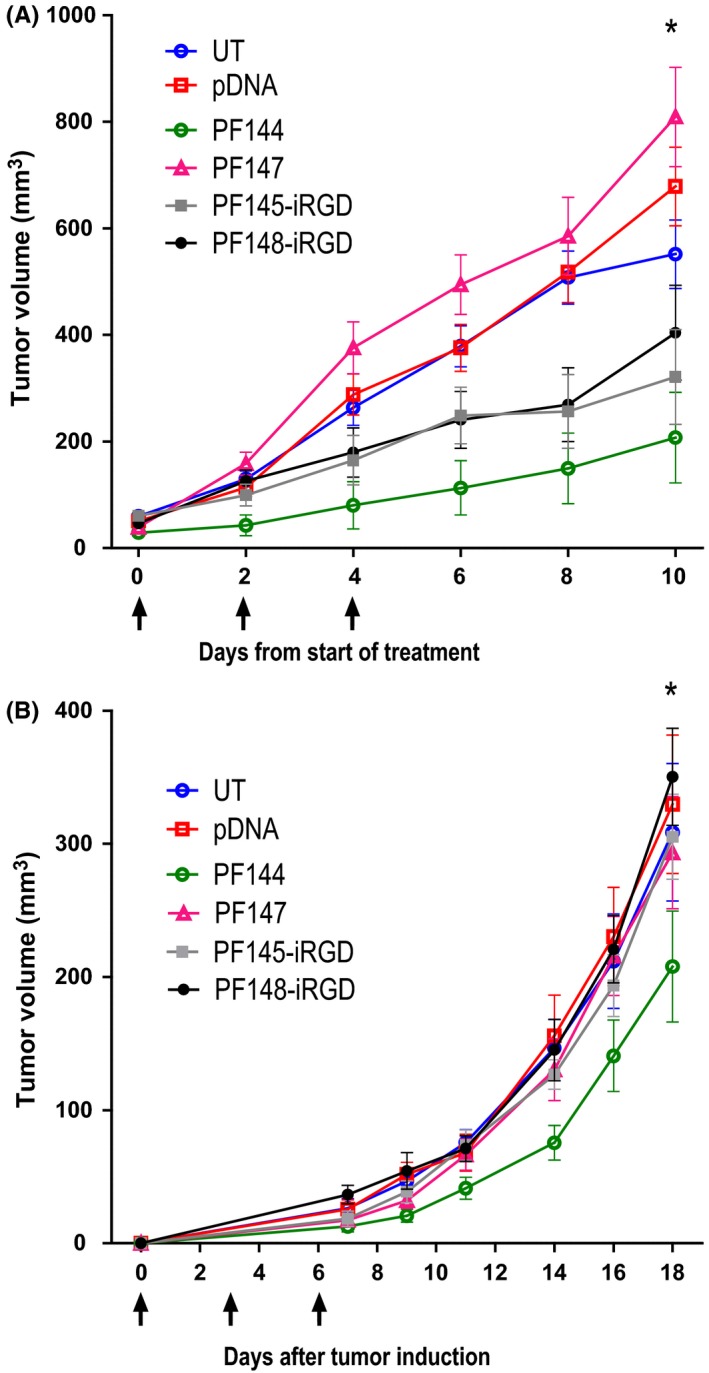
Anticancer gene therapy with PF144/pshVEGF inhibited HT1080 and 4T1 tumor growth. (A) Nude athymic mice bearing sc HT1080 tumors were sorted into treatment groups (n = 10) and administered CPP/pshVEGF complexes or pshVEGF at CR4 at a dose of 0.8 mg/kg every other day, for a total of three doses. Stars represent p‐values from Tukey post hoc comparison of tumor sizes between PF144 and pDNA groups. *P<0.05 after significant repeated measurement ANOVA (*F*(25, 375) = 2.4, *P*<0.001). (B) BALB/c mice bearing orthotopic 4T1 tumors were divided into treatment groups (n = 10) and administered CPP/pshVEGF complexes or pshVEGF at CR4 at a dose of 0.8 mg/kg every other day, for a total of three doses. Stars (*P<0.05) represent p‐values from Tukey post hoc comparison of tumor sizes between PF144 and pDNA groups after repeated measures ANOVA

Tumor therapy was evaluated with two tumor models, HT1080 and 4T1 with both expressing MMP and αvβ3 integrins,^25^, ^26^, ^27^, ^28^ and are therefore suitable for studying the potential of the activatable CPPs in tumor growth inhibition studies. The PF144/pshVEGF nanoparticles were able to significantly inhibit the tumor growth rate when compared to the group who received pshVEGF alone in HT1080 tumor‐bearing mice (Figure 3A). Complexes containing the uncleavable control peptides PF147 and PF148‐iRGD showed no effect on tumor size, as expected (Figure 3). Surprisingly, the previously observed high reporter gene induction with PF145‐iRGD/pLuc2 in reporter gene delivery assay (Figure 1C) did not translate into efficient tumor treatment. The same experiment was repeated with the 4T1 tumor model. The treatment of mice bearing orthotopic 4T1 tumors was started one day after 4T1 tumor induction, because of the aggressiveness of the 4T1 cells. The slowed tumor growth rate in the mice treated with PF144/pshVEGF was observed up to a week after the last injection (Figure 3B).

## 4 DISCUSSION

Plasmid DNA has several advantages over other oligonucleotides due to its versatility; it can encode therapeutic proteins and antisense oligonucleotides, they may contain multiple expression cassettes, and have the potential for extended transgene expression.^29^, ^30^ On the other hand, one of the disadvantages of plasmid delivery is the fact that they need to reach the cell nucleus to be effective, which is harder to achieve than cytosol delivery. Although plasmid DNA has been successfully used in combination with small molecule therapeutics,^31^, ^32^ achieving a clinical effect by plasmid delivery alone still needs to be improved. Plasmid DNA delivery by cell penetrating peptides for tumor treatment is still a challenge; CPPs need to be tumor specific to achieve safe and efficient cancer therapy through systemic delivery. Although the CPP PepFect14 (PF14) is an efficient plasmid DNA delivery vector in vivo, it has shown high accumulation in the liver and lung.^14^ The nonspecificity of PF14 was tackled in a previous study, where PF14 was modified to create MMP‐2 activatable CPP PF144, which exhibited efficient tumor specificity by reporter gene delivery.^14^ Achieving a therapeutic effect by plasmid DNA delivery with CPPs would be a big step forward in nonviral systemic cancer gene therapy.

In this study we investigated the capability of the MMP‐2 activatable cell‐penetrating peptide PF144 to inhibit tumor growth by systemic pshVEGF delivery. We observed tumor growth reduction in mice treated with PF144/pshVEGF in both the HT1080 fibrosarcoma model, and the orthotopic 4T1 breast tumor model. When looking at the tumor volume graph of the 4T1 model (Figure 3B), it can be observed that the tumor growth rate begins to restore around day 14 after tumor induction, indicating, that the effect of the transfected plasmid lasts for around a week. It has been found that some tumors adapt, and become resistant to antiangiogenic therapy, and after anti‐VEGF therapy is terminated, the tumor vasculature can quickly reestablish, leading to the regrowth of the tumor.^33^, ^34^, ^35^ The development of PF144‐based cancer gene therapy system is still early in the development process, but even though the reduction in tumor growth was modest, the system should be further developed. Anti‐VEGF therapy is commonly used as one part of a co‐therapy, so achieving a larger decrease in tumor growth should have necessitated the use of a secondary therapeutic, but since our goal was to only assess the potential of MMP‐activatable PepFects in tumor gene therapy, adding a co‐therapeutic was not optimal. One way to go forward would be by using multiple gene targets. For example, combining VEGF targeting has shown to be synergistic with HIF‐1ɑ targeting,^36^ and another interesting approach would be to use plasmid encoding IL‐12 or IL‐12‐based cytokine combination therapies.^37^, ^38^

**Figure 4:**
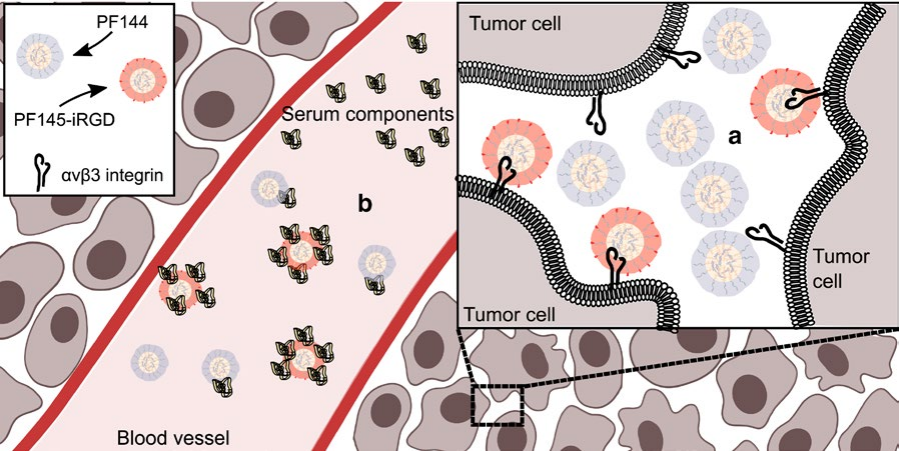
Hypothetical fate of PF144 and PF145‐iRGD complexes upon injection. According to our initial hypothesis, iRGD‐decorated PepFects should ideally concentrate near the surfaces of the cells due to binding to integrins, which should lead to more efficient linker cleavage by MMP (a). However, iRGD‐decorated PepFects showed more susceptibility to serum treatment (b), which means that a fraction of the nanoparticles get degraded, lowering the effective dose, and thus losing the advantage

We additionally studied the effect of a targeting ligand on MMP‐2‐sensitive CPPs PF144 and PF145. Since tumor‐specific αvβ3 integrins have been shown to be co‐localized with MMP‐2,^39^, ^40^ the tumor‐homing peptide iRGD was chosen for this purpose. We hypothesized, if the iRGD moiety were on the nanoparticle surface, more nanoparticles would be localized near cell surfaces due to iRGD binding to the αvβ3/5 integrins, leading to more CPPs being activated because of the higher concentration of MMP‐2 in the area. This idea is illustrated on Figure 4A. The addition of the iRGD moiety weakened the inhibitory effect of the PEG which seems to correlate adversely with the size and polydispersity of the PEG chain as indicated by cell culture experiments (Figure 1B). Since lower transfection efficiency of the PEGylated, or “non–activated” CPPs is necessary for minimization of off‐target effects, this effect leads to a reduction in tumor specificity in PF144‐iRGD, while PF145‐iRGD with a longer average PEG chain retained its tumor specificity, showing minimal luciferase induction in healthy tissues (Figure 1C). Co‐injecting the iRGD peptide with either PF144 or PF145 nanoparticles shows high reporter gene expression in the spleen that has been observed before.^41^, ^42^, ^43^ It has been hypothesized, that macrophages expressing α_v_integrins can lead to enhanced clearance of the complexes from circulation.^44^

Plasmid complexed with PF144 was more efficient in inhibiting tumor growth than PF145‐iRGD, although the latter showed high tumor accumulation tendency in reporter gene induction at the tumor site. The answer for this result could be found in nanoparticle stability experiments. PF144, the most efficient CPP in tumor growth reduction experiments also showed highest stability to serum and heparin treatment out of all the PEGylated peptides (Figure 2). There is a negative correlation between the average length of the PEG chain and the susceptibility of the CPP to serum treatment, with the CPPs with the longest PEG chains (PF145, PF145‐iRGD) showing the lowest transfection efficiency after serum treatment, while PF144 with a shorter PEG chain is more stable, and PF14 that has no PEG moiety, remains relatively unaffected by serum components. The heparin displacement assay shows the susceptibility of nanoparticles to disassembly by serum and other proteins. Again, PF144 is significantly more resistant to heparin treatment than other PEGylated CPPs tested (Figure 2B). This indicates that CPPs with iRGD decoration and longer PEG chains tend to form less condensed CPP/pDNA complexes, making them more accessible to serum components that have been shown to bind to PEG.^45^ This means, that the hypothetical advantage of the iRGD‐decorated PepFects is lost since a part of the complexes will be degraded in the bloodstream. We hypothesize that this susceptibility to serum is the reason why PF145‐iRGD performs worse than PF144 in tumor growth reduction experiments. A shorter PEG chain seems to be the best choice for constructing a tumor‐specific CPP, as we can see from PF144, but another CPP‐shielding strategy might need to be explored when modifying PepFect CPPs with iRGD; polysaccharides for example.^46^, ^47^

To conclude, we present a simple non‐viral nanoparticle system for conducting antitumor gene therapy, achieving tumor growth reduction with delivering shVEGF encoding plasmid with tumor activatable CPP PF144. We apply this strategy to suppress tumor growth as a monotherapy. Future research should be undertaken to study additional treatment strategies with our PF144/pDNA system, and explore alternative ways for CPP shielding.

## CONFLICT OF INTEREST

The authors declare no conflict of interest.

## AUTHOR CONTRIBUTIONS

K. Künnapuu, K.‐L. Veiman, L. Porosk, and K. Kurrikoff designed research; K. Künnapuu, K.‐L. Veiman, L. Porosk, and K. Kurrikoff analyzed data; K. Künnapuu, K.‐L. Veiman, L. Porosk, E. Rammul, K. Kiisholts, K. Kurrikoff performed research; K. Künnapuu wrote the paper; Ü. Langel supervised and contributed new reagents and analytic tools.
