# Supplementary material for: Tumor gene therapy by systemic delivery of plasmid DNA with cell‐penetrating peptides
Source: FASEB Bioadv. 2018 Nov 26;1(2):105–14. doi: 10.1096/fba.1026 (PMC6996304; doi:10.1096/fba.1026)
Supplement: Supplementary file 1 [file FBA2-1-105-s001.pdf]

Supplementary Figures

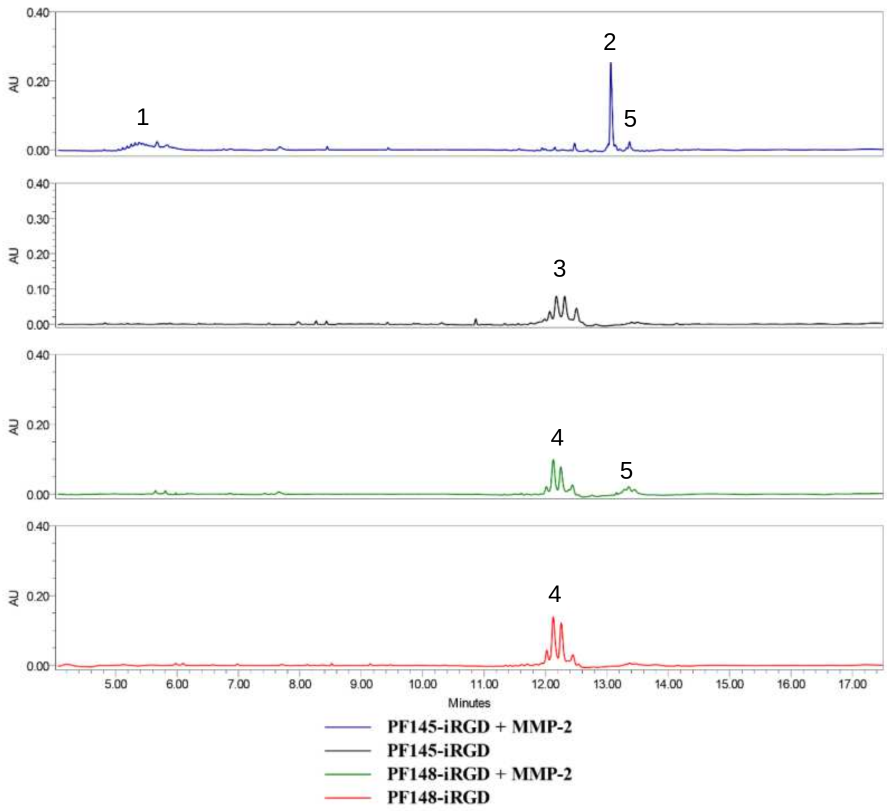

Figure S1. Cleavage assay of MMP-2 cleavable CPPs that contain iRGD. The peptides were incubated with active human recombinant MMP-2 enzyme (30 ng/µL) at 37ºC for 40 minutes, and compared to peptides without MMP-2 treatment. The mixture was then analyzed with RP-UPLC (C18 reverse phase column, eluted with 20-80% acetonitrile in water, 0.1% TFA). The figure shows the resulting chromatograms. Enzyme digestion products were confirmed by MALDI-ToF mass spectrometry. 1 – LAG-PEG1000-iRGD; 2 –Stearyl-AGYLLGKLLOOLAAAALOOLLXPLG; 3 – PF145-iRGD; 4 – PF148-iRGD; 5 – MMP-2 enzyme components. 1, 3 and 4 are composed of multiple peaks because UPLC can separate PEG containing CPPs by PEG chain length, and PEG1000 used in PF145-iRGD and PF148-iRGD is polydisperse.

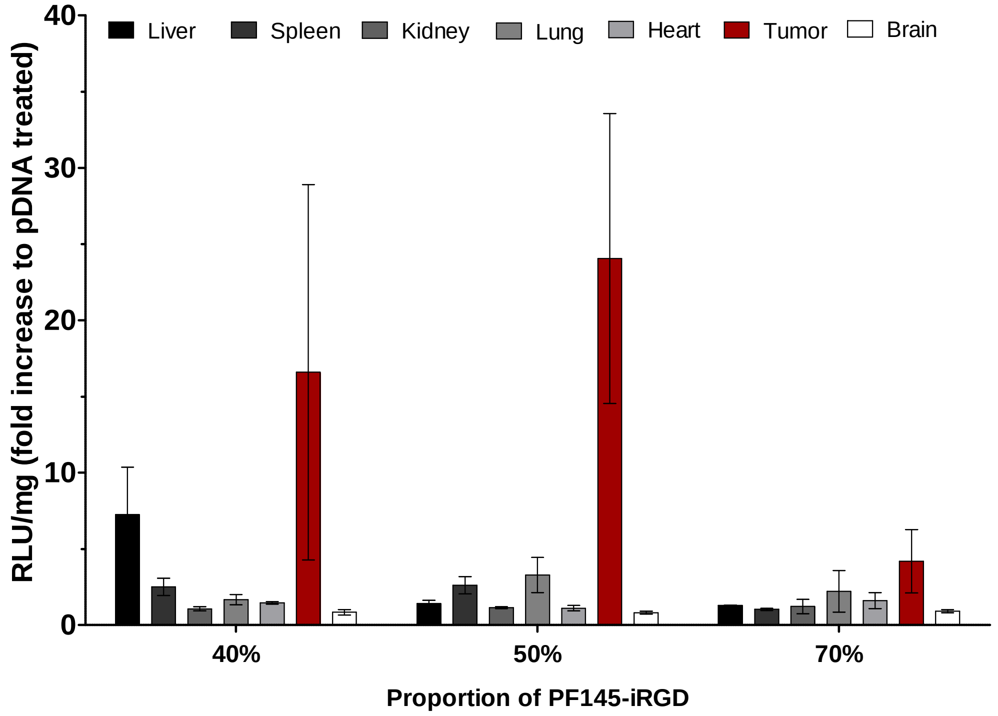

Figure S2. The ability of PF145-iRGD/pLuc2 complexes (CR4, 20µg) to induce gene expression in mice bearing subcutaneous Neuro2a tumors. Different pegylation rates were tested, meaning that the amount of PF145-iRGD varied from 40% to 70%, the rest of the peptide in the complex consisted of PF14. Complexes were administered intravenously via tail vein. Tissues were harvested 24 hours after the injections and their luciferase content was measured. N=3 in each group.

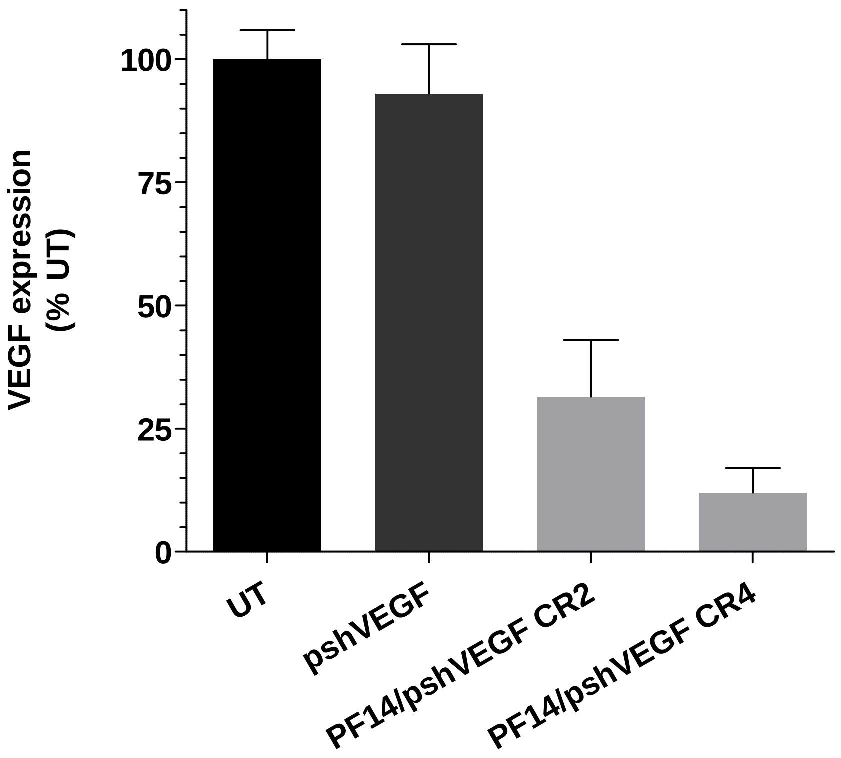

Figure S3. Validation of shVEGF encoding pDNA *in vitro*. Gene expression levels of VEGF were measured 48h after HT1080 cell transfection with PF14/pshVEGF complexes at 2 different charge ratios. For that, cells were lysed and RNA extracted using Trizol, followed by qRT-PCR. Data was analysed using $2^{-\Delta\Delta CT}$ method.

Supplementary methods

Peptide cleavability by MMP-2

To assess the cleavability of iRGD-modified CPPs by MMP-2, the peptides were incubated with recombinant MMP-2 (0.1 mg/ml, Calbiochem, Germany) in MQ water for 40 minutes at 37°C and analyzed with UPLC.

pshVEGF validation

To validate the shVEGF encoding pDNA, gene expression levels of VEGF were measured 48h after HT1080 cell transfection. The cells were transfected according to cell transfection protocol detailed in the Methods section of the paper. Briefly, HT1080 cells were seeded onto 24-well plates, and peptide/pDNA complexes at CR2 and CR4 was added to cells growing in full media when they reached 50% confluency. The transfection mixture made up 1/10 of the

total cell medium volume during the time of transfection. For hours after adding the CPP/pDNA complexes, an additional 1 ml of fresh full medium was added to the cells. 48 hours after cell transfection, the cells were lysed and RNA extracted using Trizol reagent (Invitrogen) and cDNA synthesized using Superscript III revertase (Invitrogen). qRT-PCR was conducted using EvaGreen qPCR Supermix reagent (Solis BioDyne) and data was analysed using $2^{-\Delta\Delta CT}$ method.
